# Distribution of Axial Length before Cataract Surgery in Chinese Pediatric Patients

**DOI:** 10.1038/srep23862

**Published:** 2016-03-29

**Authors:** Haotian Lin, Duoru Lin, Jingjing Chen, Lixia Luo, Zhuoling Lin, Xiaohang Wu, Erping Long, Li Zhang, Hui Chen, Wan Chen, Bo Zhang, Jinchao Liu, Xiaoyan Li, Weirong Chen, Yizhi Liu

**Affiliations:** 1 State Key Laboratory of Ophthalmology, Zhongshan Ophthalmic Center, Sun Yat-sen University, Guangzhou, Guangdong, 510060, People´s Republic of China

## Abstract

Axial length (AL) is a significant indicator of eyeball development, but reports on the overall status of axial development in congenital cataract (CC) patients and its relationship with patient demographics, such as age, sex, and laterality, are rare. We prospectively investigated the AL of 1,586 patients ≤18 years old and undergoing cataract surgery in China from January 2005 to December 2014. Of these 3,172 eyes, a logarithmic correlation between AL and age in CC patients was calculated, and an age of approximately 2 years was found to be a turning point in the growth rate of AL. A considerable variation was observed in CC patients of the same age. Furthermore, 2–6 years old boys had longer AL than girls. The AL of affected eye in unilateral patients was longer than that of the contralateral eye in 2–6 years age group and longer than that of eye in bilateral CC patients in all age groups. These findings indicate that the development of the length of eyeballs in CC patients is influenced by multiple factors in addition to age. A full understanding of the distribution of AL may provide a useful reference for judging the timing of surgery in CC patients.

Axial length (AL) is a significant indicator of eyeball development and correlates with heredity^1^, development^2^,^3^, nutrition, and other environmental factors. Congenital cataract (CC) is a primary cause of pediatric cataract that also affects eyeball development, and the mechanisms that underlie the pathological changes to AL include defocusing^4^,^5^,^6^,^7^ and form deprivation^8^,^9^. Previous studies have revealed that the AL in pediatric CC patients is different from AL in healthy children^10^, and varies with different types and duration of cataract. However, reports on the overall status of axial development in CC patients and its relationship with patient demographics, such as age, sex, and laterality, are rare. Furthermore, the AL of CC patients is not only a crucial indicator of eyeball development and treatment prognosis, but also a vital contributor to intraocular lens (IOL) power calculation^11^. To reduce the effect of developmental opacity of the lens and vitreous on the accurate measurement of AL, contact A-scans, which show relatively good accuracy and maneuverability, are currently the most frequently used method for AL measurement before surgery in pediatric CC patients^12^,^13^. Most of the previous studies related to CC in clinical practice have proposed a treatment plan solely according to the age of the patient^14^, regardless of the actual development of AL, which may result in better and more precise treatment. Indeed, this approach has resulted in significant variability in the treatment effects observed in pediatric CC patients. Data on the overall distribution of AL before cataract surgery is critical for judging the timing of surgery and for improving the accuracy of postoperative IOL power in CC patients.

To our knowledge, few investigations have examined patient demographics and the distribution of AL before cataract surgery in a large cohort of Chinese pediatric patients with CC. In this prospective, large sample study, we aimed to investigate the overall status of axial development in CC patients younger than 18 years old and the relationship between AL development and patient demographics, including age, sex, and laterality, and our results provide a useful reference for the accurate timing of IOL implantation and CC treatment.

## Results

### Patient demographics

**Figure 1:**
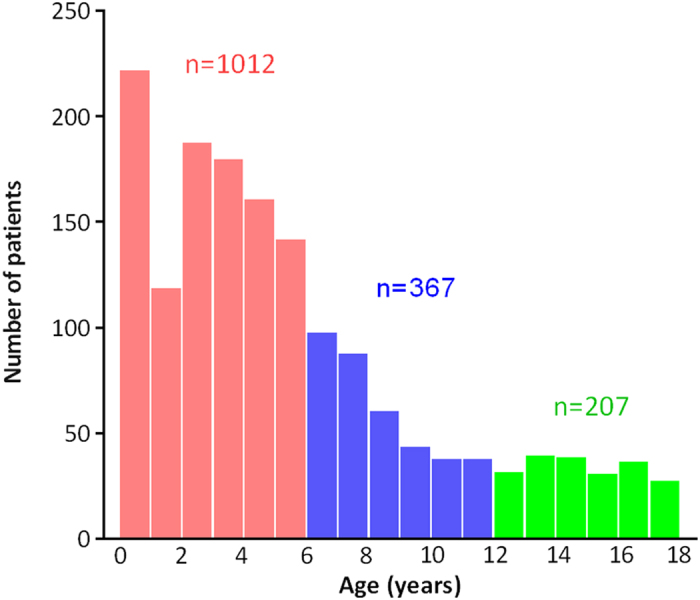
The distribution of CC patients with different ages. Nearly 63.81% (1012/1586) of CC patients were younger than 6 years old, and the number of patients decreased with age.

Complete AL data were collected for both eyes from a total of 1,586 pediatric CC patients (3,172 eyes) who were enrolled from January 2005 to December 2014. Most of these patients were confirmed residents from south China. Bilateral cataract patients represented a fairly large proportion (68.85%, 1092/1586) of the study population, and the remaining 31.15% (494/1586) of the subjects were unilateral patients. The ratio of boys to girls was 1.41 (928: 658), and the mean age of these subjects was 68.86 ± 54.90 months (95%CI 66.16–71.57). The constitution of all analyzed subjects in different ages was shown in Fig. 1, and the patients aged 0–6 years old constituted the highest patient proportions. The right eye was arbitrarily selected to represent the AL of bilateral individuals, because no difference of AL of two eyes in bilateral CC patients were found by paired T-test (t = 1.353, P = 0.176).

Among all of the analyzed CC patients, 82.16% (1303/1586) of them were undergoing cataract extraction and IOL implantation, with a mean age of 81.05 ± 52.27 months old, and the remaining 17.84% (283/1586) were undergoing cataract extraction and had a mean age of 12.74 ± 21.87 months old. The AL of the patients treated with the different surgical procedures mentioned above were significantly distinct: 22.90 ± 1.99 mm vs. 19.81 ± 2.01 mm, respectively.

### Relationships between AL and age, gender, laterality

**Figure 2:**
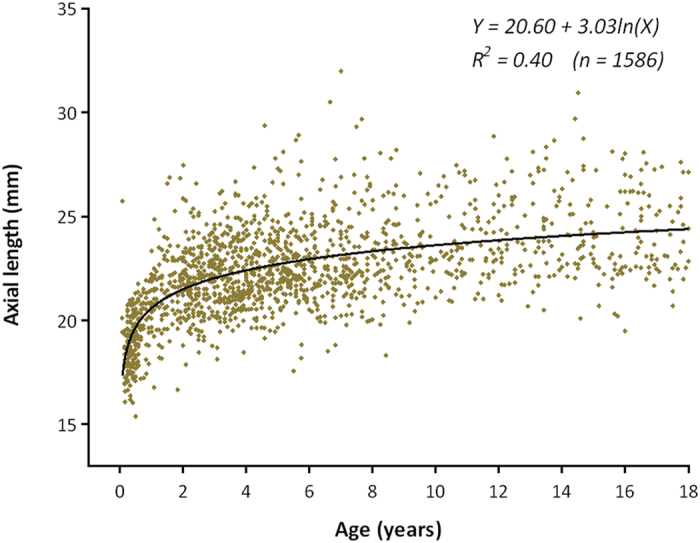
Scatterplot of axial length by patient’s age and fitted curve for cataractous eyes of patients. Y: axial length; X: age in years.

The logarithmic correlation of AL to age in CC patients ≤18 years old was presented in Fig. 2. The AL elongated more significantly in younger patients: a trend of faster growing before age 2 years with a plateau thereafter can be hypothesized.

**Figure 3:**
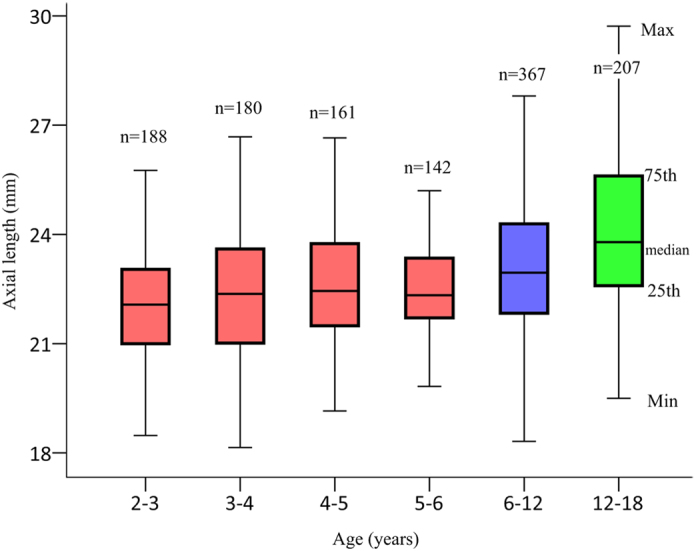


Although AL measurements were age dependent and plateau in children >2 years old, considerable variation was observed in those CC patients of the same age (Fig. 3). The quartile deviations (differences between the 75^th^ and 25^th^ percentiles) of the 6 age subgroups were, from youngest to oldest, 2.05 mm, 2.59 mm, 2.27 mm, 1.66 mm, 2.47 mm and 3.03 mm, respectively.

Table 1 showed that the AL of boys aged of 2–6 years was statistically longer than that of girls of the same age in both bilateral and unilateral cataract group.

The AL of affected eyes in unilateral CC patients was longer than that in bilateral CC patients, as shown in Table 2. Table 3 showed that the longer AL in the affected eye than in the contralateral eye of unilateral CC patients was mainly distributed in patients 2–6 years old.

## Discussion

AL is not only a crucial indicator of eyeball development but also a vital contributor to calculating IOL power in pediatric CC patients. Data on the overall distribution of AL before cataract surgery is critical for judging the timing of surgery and improving the accuracy of postoperative IOL power. However, published reports of axial development in pediatric CC patients are limited to studies with small sample sizes^15^,^16^ or studies of specific aspects, such as age or laterality. To the best of our knowledge, this is the largest study to provide information on the overall status of axial development and its relationship with patient demographics, including age, sex, and laterality, in a large cohort of Chinese pediatric CC patients (3,172 eyes of 1,586 CC patients) before cataract surgery. The Zhongshan Ophthalmic Center (ZOC) is one of the best, largest, and oldest eye hospitals that uses representative, current CC treatment methods in China^17^,^18^. All of the participants were confirmed to be from south China and this group of people represented the demographics and distribution of AL in CC patients in this region. Similar to what has been seen in other studies, pediatric patients presenting for CC surgery in this study were predominantly male and had bilateral cataracts^19^,^20^, which may be attributed to the traditional preference for sons in China or to gender-related genetic mechanisms that have previously been described^21^. The mean age of the CC patients presenting for the surgery in this study was 68.86 ± 54.90 months. However, most CC patients can be identified and treated within 100 days of birth in many developed countries because of national screening procedures^22^, indicating that delayed presentation to the hospital and late surgical treatment are very common in China. The ALs of patients treated with different surgical procedures (cataract extraction with/without IOL implantation) were distinct, which may be largely due to differences in age at the time of surgery (81.05 ± 52.27 months vs. 12.74 ± 21.87 months).

AL is known to increase with age, and the eye changes in size early in life and during childhood in healthy children, concomitant with height and overall development^2^,^23^. A logarithmic correlation between AL and age was also found in CC patients in the first 42 months of life in a retrospective study^15^. In this prospective study, similar findings were obtained in 1,586 CC patients who were less than 18 years old, likely resulting from the sharing of common biological pathways during overall growth and AL growth^2^. Furthermore, a remarkable rise in the AL of CC patients ≤2 years old was observed, which plateaued in older children, and this finding is partially consistent with a previous study showing that AL changes rapidly in the first 18 months of life in the non-cataractous eyes of unilateral CC patients^24^. In addition, AL is of vital importance for IOL power calculations in CC patients. The long-term results for CC after primary IOL implantation remains controversial in children less than 2 years of age, who experience rapid elongation in AL. Many researchers oppose primary IOL implantation in very young children, complaining a combination of greater prediction error^25^, future myopic shift^26^, unmatched IOL size^16^, and a higher frequency of postoperative complications^27^. Infant Aphakia Treatment Study Group (IATS) have proposed that delaying IOL implantation until later years after most of the growth in AL has occurred benefits for selecting a more appropriate IOL power for implantation^28^. The rapid growth rate of AL in CC patients less than 2 years old observed in this study provides a useful reference, with exact data, in terms of AL, for objections to implanting IOL in very young CC children. While some small case series have reported relatively good visual outcomes following unilateral IOL implantation during infancy^29^,^30^, more recently, well-controlled myopic shift and reasonably good visual acuity after primary IOL implantation were reported in a retrospective study in India of children who underwent surgery for CC when they were less than 2 years old^16^. However, this clinic-based study was likely not representative because it involved only 13 participants and showed severe selection bias. Thus, determining long-term results following primary IOL implantation in CC patients who are less than 2 years old will require additional large-scale investigations^31^.

A positive correlation between AL and age is generally accepted, but there is very little published information that specifically describes the distribution of AL in CC patients of the same age. Although placing an IOL in patients more than 2 years old is a relatively better option according to the growth rate of AL, considerable variations of AL observed in CC patients (2–18 years old) of the same age in the present study should also be taken into account. The quartile deviations of AL of patients of the same age were 1.66–2.59 mm in patients aged of 2–6 years, and likely resulted in 7.77 diopters (D) of refractive error (3 D per millimeter), at most. Most previous studies related to CC clinical practices have supported a treatment plan based solely on the age of the patient^14^; however, an accurate measurement of AL should be taken into consideration to achieve better and more precise results, even in patients of the same age.

The increase in AL follows the overall growth and development of body size, which is regulated largely by the sex steroids (androgens and estrogens). Indeed, sex-linked differences in the AL of infants and children have been reported in the literature. Isenberg *et al.*^32^ found that the eyes of male infants grow faster than those of female infants, and Larsen *et al.*^33^ and Trivedi *et al.*^34^ noted shorter mean AL in girls than in boys in both non-cataractous eyes and cataractous eyes. However, Capozzi *et al.*^15^ reported no difference in AL according to sex. In the present study, we found that the AL in boys was significantly longer than that in girls in 2–6 years old age subgroup. One possible explanation for this difference is the close correlation between AL and head size, which has been previously reported^35^,^36^; for example, boys have a larger head circumference than girls before 5 years of age, according to the data published on the website of the World Health Organization (WHO).

The impact of cataracts on the development of the eye is not related only to the age and sex of the patient but also depends largely on laterality. A shorter AL was previously noted in young pediatric bilateral CC patients (≤42 months old) compared to the affected eyes of patients with unilateral CC involvement^15^. Similar findings were also revealed in the present study. However, Trivedi *et al.*^34^, in contrast to our results, reported that cases of bilateral cataracts had a longer AL than those with a unilateral cataract in patients younger than 60 months of age, but a shorter AL than the eyes with a unilateral cataract in patients older than 60 months of age. One possible explanation for these differences is the race of the participants (Caucasian or African-American vs. Chinese), which may contribute to distinct outcomes because race is known to influence biometry results in children. Differences in the study type, grouping situation and analysis of sample size could also account for these discrepancies. In patients with unilateral cataract, the cataractous eye was reported to have a significantly shorter AL than the contralateral normal eye in CC patients who were less than 7 months old^9^. Capozz *et al.*^15^ reported no difference in AL among unilateral CC patients who were less than 42 months old. However, results of the present study show that cataractous eyes have longer AL than non-cataractous eyes in patients with a unilateral cataract in different age subgroups, and this is partially in line with the finding of Trivedi and associates^34^. Together, these findings indicate that the AL of unilateral CC patients is likely age-dependent and related to the duration of form deprivation. The exact relationship between the AL of eyes in CC patients with differences in laterality is affected by numerous factors and remains to be further studied.

The results of this study must be assessed within the context of its limitations. First, the contact A-scan measurements used in this study likely yielded shorter AL measurements than those from immersion A-scans in pediatric eyes^12^. However, this deviation was minimized by using the same apparatus for all subjects; moreover, contact A-scans remains the most appropriate tool for CC patients who are unable to cooperate for long periods, and these scans are an easier tool for examiners with large workloads to use. Second, the data do not provide a complete representation of the population and only relate to surgical patients for whom we gathered complete data regarding AL in the best eye center in south China, which receives admissions that are biased toward patients who are unsuitable for surgery and who present with complicated or serious diseases. Despite these limitations, the results of this study describe the overall distribution of AL before cataract surgery in Chinese pediatric patients because of its large sample size (3,172 eyes of 1,586 patients) and the representativeness of its population in south China.

In conclusion, pediatric patients who presented for surgery and were included in this study were predominantly male and had bilateral cataracts. The AL of CC patients with different ages, genders and laterality was distinct, indicating that the development of the length of eyeballs in CC patients is influenced by multiple factors in addition to age. A full understanding of the distribution of AL and accurate measurements of AL will likely provide a useful reference for judging the timing of surgery and improving the accuracy of postoperative IOL power achieved in CC patients.

## Methods

### Subjects

This study was a prospective large cohort research, which was included as one of our series ongoing studies of the Childhood Cataract Program of the Chinese Ministry of Health (CCPMOH). Patients with CC who were undergoing cataract extraction with/without IOL implantation were consecutively recruited during pre-operation screening at the ZOC, Guangdong, China, from 1 January 2005 to 31 December 2014. The medical history and systematic history of each of the patients were carefully learned. The CC in this study was defined as a cataract caused by heredity or developmental disorders. All patients were examined and confirmed as a CC victim by at least three experienced ophthalmologists before the cataract surgical procedure, which included a detailed slit-lamp examination and thorough ophthalmoscopy through dilated pupils. Recruited CC patients were no more than 18 years old and were undergoing cataract extraction with/without IOL implantation. Patients with congenital intrauterine infection cataract (such as rubella cataract), complicated cataract (such as uveitis cataract), metabolic cataract (such as tetany cataract), and traumatic cataract were excluded. Patients with severe ocular disease, infection, cough, or other systemic diseases likely to affect the AL examination and surgical procedure were also excluded. To obtain a better analysis, patients were divided into several groups according to age, sex and laterality, when necessary.

### A-scan examination

A contact A-scan (B-SCAN-Vplus/BIOVISION, Quantel Medical, France) was used to obtain the AL measurements before surgery. The A-scan unit was equipped with a 10 MHz transducer probe, and the velocities were set as follows: 1,641 m/s for the cornea and lens and 1,532 m/s for the aqueous and vitreous. All patients were tested by the same examiner, and some of the patients who were unable to actively cooperate were sedated using 10% chloral hydrate (0.8 ml/kg, oral or rectal administration). Applanation ultrasound was performed after instillation of one drop of topical anesthetic (0.5% Alcaine, Alcon, USA) to the lower conjunctiva. Each eye was measured 10 times, and the mean measurements were used. This study was approved by the Human Research Ethics Committee of the ZOC at Sun Yat-sen University. All procedures adhered to the tenets of the Declaration of Helsinki, and written informed consent was obtained from at least one parent of each patient.

### Statistical analysis

Statistical analysis was performed using the Statistical Package for the Social Sciences (SPSS ver. 19.0, Chicago, IL, USA). Absolute frequency (n) and relative frequency (%) were used to describe qualitative variables; mean and standard deviation (mean ± SD) were used for age, AL, and other quantitative variables of CC patients. The Kolmogorov-Smirnov test was used to evaluate the normality of the distribution for all variables. The T-test for independent samples was used to analyze the difference of AL between patients with different age, gender and laterality, and the paired T-test was used to evaluate the difference of AL between the affected eye and the fellow eye in unilateral CC patients. A P value < 0.05 was considered statistically significant.

## Additional Information

**How to cite this article**: Lin, H. *et al.* Distribution of Axial Length before Cataract Surgery in Chinese Pediatric Patients. *Sci. Rep.* **6**, 23862; doi: 10.1038/srep23862 (2016).

## Figures and Tables

**Table 1:** Comparison of the axial length between girls and boys by adjusting age and laterality of the cataract.

	0–2 Y	2–6 Y	6–12 Y	12–18 Y	0–18 Y
Bilateral					
Girls	19.94 ± 2.04	21.94 ± 1.69	23.04 ± 2.08	24.16 ± 1.84	22.06 ± 2.32
	(n = 98)	(n = 177)	(n = 101)	(n = 63)	(n = 439)
Boys	19.68 ± 1.85	22.48 ± 1.55	23.31 ± 1.96	23.70 ± 2.00	22.12 ± 2.31
	(n = 163)	(n = 262)	(n = 144)	(n = 84)	(n = 653)
t	1.06	−3.46	−1.04	1.43	−0.40
P	0.290	**0.001**	0.300	0.155	0.692
Unilateral					
Girls	20.86 ± 1.95	22.35 ± 1.84	22.83 ± 1.92	24.91 ± 2.53	22.50 ± 2.26
	(n = 40)	(n = 101)	(n = 51)	(n = 27)	(n = 219)
Boys	21.34 ± 1.82	22.88 ± 1.73	23.53 ± 1.98	24.90 ± 2.03	23.06 ± 2.07
	(n = 40)	(n = 131)	(n = 71)	(n = 33)	(n = 275)
t	−1.14	−2.25	−1.94	0.22	−2.87
P	0.259	**0.025**	0.054	0.983	**0.004**

The data are presented as the means ± standard deviation (SD). Bold data are significant at P < 0.05 (independent sample T-test); Y: years.

**Table 2:** Comparison of the axial length in eyes of bilateral and unilateral CC patients by adjusting age and gender.

	0–2 Y	2–6 Y	6–12 Y	12–18 Y	0–18 Y
Girls					
Bilateral	19.94 ± 2.04	21.94 ± 1.69	23.04 ± 2.08	24.16 ± 1.84	22.06 ± 2.32
	(n = 98)	(n = 177)	(n = 101)	(n = 63)	(n = 439)
Unilateral	20.86 ± 1.95	22.34 ± 1.84	22.83 ± 1.92	24.91 ± 2.53	22.50 ± 2.26
	(n = 40)	(n = 101)	(n = 51)	(n = 27)	(n = 219)
t	−2.44	−1.88	0.59	−1.39	−2.32
P	**0.016**	0.062	0.557	0.171	**0.021**
Boys					
Bilateral	19.68 ± 1.85	22.48 ± 1.55	23.31 ± 1.96	23.70 ± 2.00	22.12 ± 2.31
	(n = 163)	(n = 262)	(n = 144)	(n = 84)	(n = 653)
Unilateral	21.34 ± 1.82	22.88 ± 1.73	23.53 ± 1.98	24.90 ± 2.03	23.06 ± 2.07
	(n = 40)	(n = 131)	(n = 71)	(n = 33)	(n = 275)
t	−5.11	−2.29	−0.77	−2.91	−5.85
P	**<0.001**	**0.023**	0.440	**0.004**	**<0.001**

The data are presented as the means ± standard deviation (SD). Bold data are significant at P < 0.05 (independent sample T-test); Y: years.

**Table 3:** Comparison of the axial length of the affected eye and fellow eye in unilateral cataract patients.

Age (Y)	n	Affected Eye (mm)	Fellow Eye (mm)	t	P
0–2	80	21.10 ± 1.89	21.07 ± 1.39	0.19	0.850
2–6	232	22.65 ± 1.79	22.13 ± 0.99	4.85	**<0.001**
6–12	122	23.24 ± 1.98	22.96 ± 1.27	1.60	0.113
12–18	60	24.90 ± 2.25	24.46 ± 1.20	1.45	0.153
0–18	494	22.82 ± 2.17	22.45 ± 1.50	4.70	**<0.001**

The data are presented as the means ± standard deviation (SD). Bold data are significant at P < 0.05 (paired sample T-test); Y: years.
